# Current Evidence and Considerations for Psychological Support Interventions for Fathers in the Neonatal Intensive Care Unit

**DOI:** 10.3390/ijerph23020144

**Published:** 2026-01-23

**Authors:** Alyssa R. Morris, Anahit Sarin-Gulian, Catherine Mogil

**Affiliations:** 1 David Geffen School of Medicine, University of California, Los Angeles, CA 90095, USAcmogil@mednet.ucla.edu (C.M.); 2 Center for Perinatal and Infant Research, Rady Children’s Health, Orange, CA 92868, USA

**Keywords:** newborn infant, neonatal intensive care units, fathers, paternity, caregivers, integrated delivery of health care, psychological stress, mental health, psychosocial intervention, policy

## Abstract

There is a lack of focus on psychological support for fathers in Neonatal Intensive Care Units (NICUs), both in research and practice, with fathers receiving far less support from NICU providers as compared with mothers. This article aims to discuss the current literature and limitations related to providing psychological support to fathers in the NICU and proposes short- and long-term efforts for improving psychological care for NICU fathers. We conducted a narrative literature review to summarize interventions for supporting fathers in the NICU, including emotional support, educational support, social support, family-integrated care, and multi-component interventions. While initial work is promising, there are major limitations. Very few studies have examined interventions specific to providing support to fathers in the NICU, and little work has investigated differences in the support needs and responses to interventions for NICU fathers as compared with mothers. Fathers have historically been overlooked in the NICU. Given the growing recognition of paternal mental health challenges and their impact on infant development, there is a pressing need for efforts aimed at supporting fathers in the NICU. Efforts must consider system structure, policy, multidisciplinary training, and implementation protocols to improve the quality of care provided to NICU fathers.

## 1. Introduction

Fathers have historically been the “forgotten parent,” with the vast majority of literature on parenting, particularly during the perinatal period, focusing on mothers [1]. When it comes to research on parents of hospitalized infants, publications focused on supporting fathers are even more narrow. Despite the lack of consideration of fathers, paternal involvement and mental health needs have been increasingly recognized as important, not only for fathers themselves, but also for their baby and coparent [2,3]. There has been an increasing recognition of the prevalence of paternal postpartum depression and other paternal mental health challenges, and research has shown that paternal mental health has an impact on a number of aspects of infant development [4,5].

In the Neonatal Intensive Care Unit (NICU), providers often attune to the physical and emotional needs of mothers, with fathers receiving less support [3,6,7,8]. However, fathers are in desperate need of care. Studies have found that fathers of infants in the NICU are at increased risk of developing postpartum depression and trauma- and stress-related disorders as compared with fathers of healthy infants [9], with up to one third of NICU dads experiencing depressive, anxiety, stress, and posttraumatic stress symptoms [10]. Additionally, research has found that while mothers’ depressive symptoms tend to improve over the course of the NICU stay, fathers’ depressive symptoms do not [10,11]. While immediate mother-infant interaction is typically prioritized in non-complicated births, the birthing experience and initial parental involvement differ significantly when babies go to the NICU. Fathers may have more opportunities to be involved directly after birth, as compared with birthing mothers [2], often due to complications related to maternal medical care needs. Thus, paternal involvement and wellbeing are particularly important for infants hospitalized in the NICU.

Research on psychological support for NICU fathers, particularly Black fathers and other underrepresented racial and ethnic backgrounds [12,13], continues to lag far behind research on support for mothers [14]. It is vital to explore gender identity and role differences in parents, as it is unknown if interventions suitable for mothers are as effective for fathers. Additionally, community-based interventions that focus on parenting more broadly, even those that touch on fatherhood, may not be relevant, effective, or even feasible in the hospital setting [15]. A recent systematic review and meta-analysis suggests that while there are very few studies exploring mental health interventions for NICU fathers, initial work is promising [16]. There is a clear need to develop and evaluate programs that support fathers in the NICU context. This paper provides a brief discussion of the current literature, limitations to psychological support for NICU fathers, and considerations for system-level change to support paternal mental health. Perspectives discussed in this manuscript integrate our interpretation of the literature with our observations through clinical experience supporting the mental health needs of NICU families.

## 2. Methods

This manuscript provides a narrative review of the literature on psychological support interventions for fathers in the NICU and discusses related challenges and constraints based upon our clinical experience in the field. We were specifically interested in work that explored the impact of a mental-health-related intervention on fathers’ wellbeing (i.e., stress, anxiety, depression, and trauma symptoms). We excluded articles that focused solely on interventions aimed at increasing fathers’ presence or infant holding, including papers specifically examining the effects of kangaroo care. Papers of interest included those that incorporated a counseling component, either emotional or informational. It is important to note that this review is not systematic or scoping in nature. This manuscript started as a basic literature search to find potential mental health interventions for implementation with NICU fathers at UCLA Ronald Reagan Medical Center and UCLA Santa Monica Medical Center. Through this process, we recognized value in disseminating the themes we observed, along with their intersections with our clinical experiences and challenges. We begin with a summary of our narrative review of psychological support interventions for fathers in the NICU and then discuss the challenges and constraints we’ve observed in our clinical work with this population.

## 3. Intervention Research with NICU Fathers

Although the importance of the fatherhood role is garnering more attention, there remains limited research exploring interventions specifically helpful for fathers of infants in the NICU. Meta-analyses, systematic, and scoping reviews in this area have highlighted the dearth of research exploring interventions specific to fathers in the NICU [3,15,17,18]. A comprehensive review published in 2021 aimed to describe interventions for supporting fathers and found only seven studies exploring interventions for NICU parents that included fathers. Of those, only one specifically assessed an intervention for fathers, whereas the other six explored interventions aimed at parents more generally [15]. In an even more recent meta-analysis of the effectiveness of psychosocial interventions on parents of preterm infants, authors found only two studies specific to paternal support, as compared with 10 studies assessing interventions for mothers. They found an additional six intervention studies explored interventions for parents generally, with only three of these reporting results separately for fathers and mothers, allowing for insights into the paternal experience [15]. While not specific to mental health outcomes, a scoping review focused on recommendations for supporting NICU fathers identified 18 articles that provided guidance on supporting an inclusive NICU environment that incorporates fathers [3]. The dearth of research in this area reflects the real-world lack of support for NICU fathers. Fathers receive less attention and support not only from NICU staff [6,7], but also receive far less support from other NICU parents as compared with NICU mothers [19]. This highlights a need for concerted efforts in supporting NICU fathers.

### 3.1. Differences Between Mothers and Fathers

While there are limited studies exploring interventions for fathers, a 2024 meta-analysis found that psychosocial interventions aimed at supporting NICU parents were effective at reducing symptoms of stress, depression, anxiety, and traumatic stress, and that there were no significant differences in these outcomes between mothers and fathers [15]. However, other research suggests that there are noteworthy differences in the emotional experience of NICU fathers that may limit the generalizability of interventions across parents. A systematic review of interventions aimed at increasing paternal involvement in the NICU identified several differences in the effects of interventions between mothers and fathers [2]. For example, they found that maternal and paternal baseline stress reports differed, with fathers reporting lower self-confidence and stress than mothers [2]. In another study, researchers found differences in the emotional experience of the NICU for mothers and fathers, noting that fathers felt intense internal distress but reacted by hiding their emotions in an effort to protect themselves from emotional pain [19]. Further, NICU fathers feel pressure to be strong for their partner and child, and subsequently experience emotional overwhelm in private [3,19]. Additionally, the stressors contributing to anxiety and depression in fathers appear to be different from those of mothers, with fathers struggling with financial pressures, other household responsibilities, and balancing work and NICU time [20]. Studies exploring the coping strategies used by NICU fathers highlight the use of problem-oriented [19,21] and emotion-based coping [22], which may vary based on culture. Given the differences in paternal emotional experiences and responses, it is essential for interventions to target the unique differences and needs of paternal mental health. This should include considerations for how mental health challenges may manifest in men as compared with women (i.e., men exhibit more externalizing symptoms, women exhibit more internalizing symptoms), as well as the well-established reluctance of men to report mental health symptoms [23].

### 3.2. Existing Interventions for Fathers

There are a limited number of studies that have examined interventions designed specifically to support fathers in the NICU. Of these, one described a peer support group model at the Royal Women’s Hospital in Melbourne, Australia, with promising reports of improved father involvement and father-infant bonding [24]. Additionally research out of Taiwan explored [25,26] the impact of an educational intervention for NICU fathers, finding decreased paternal distress, increased father-infant attachment and fathering ability, and higher perceptions of nursing support [25,26]. These studies are a promising step as the field continues to develop interventions to support NICU fathers.

While there are a very limited number of studies that have explored interventions designed specifically for fathers in the NICU, literature exploring support interventions for NICU parents more generally provides additional insight into the potential value of psychological interventions for fathers. Findings from the above studies will be discussed in further depth, along with other interventions used in work with NICU fathers, which we have broken down into categories roughly based on Ocampo et al. [18]: emotional support, educational support, social support, family-integrated care, and multi-component interventions.

#### 3.2.1. Emotional Support Interventions

A meta-analysis conducted by Chan & Shorey [15] found that emotional support, which includes interventions that provide opportunities for parents to share their feelings in a safe and supportive environment, was effective in minimizing symptoms of distress among NICU parents. As noted above, a descriptive study of a peer-based emotional support intervention customized for the needs of fathers was developed at the Royal Women’s Hospital in Melbourne, Australia. Thomson-Salo et al. [24] described their experience implementing a peer support group for fathers with a baby in the NICU. Co-facilitated after work hours by an embedded infant mental health clinician and male neonatologists, the group focused on promoting mutual support among fathers, including discussions of the traumatic birth and NICU journey, personalities of their infants, unexpected joy of skin-to-skin, and sadness about the lack of privacy during precious moments. Reports from participants and staff highlighted the benefits of paternal involvement on father-infant bonding and the father-staff relationship [20]. This initial work is promising, and more empirical research will be essential to establish the effectiveness of emotional support in the NICU setting for fathers.

One of the few empirical studies on the effectiveness of emotional support-focused intervention in the NICU to report data on fathers comes out of Sweden. This study explored the effectiveness of a support program on maternal and paternal stress. The intervention was carried out by bedside nurses and was developed based on parents’ experiences and needs, family-centered care, and person-centered communication. The intervention included four dialogs related to the parents’ NICU experience (i.e., birth experience, connection to infant, parents’ reactions, and reflection of experiences during the NICU stay). Interestingly, this intervention was found to decrease maternal, but not paternal stress [27], highlighting that emotional support-focused interventions may need to be modified for use with fathers.

While there are limited studies on emotional support specifically for fathers, researchers have explored various methods of providing emotional support for parents more generally (i.e., samples including both mothers and fathers grouped together). Two studies found that NICU peer support groups focused on parental empowerment and social connection were useful in decreasing feelings of isolation [28], reducing parent depressive symptomatology, and increasing parental self-efficacy [29]. Both of these studies used methodologies that limited the conclusions that could be drawn, in that the first was a descriptive case study [28], and the second utilized a comparison group drawn from people who registered but never showed up for any of the six sessions [29]. Interestingly, one of the most rigorous studies of peer-based support for NICU parents to date—a pre-registered, randomized control trial of 300 families in a United States (Washington D.C.) NICU—did not produce improvements for the intervention group beyond those of controls [30,31]. This study examined the value of peer support for families over the first 12 months following discharge from the NICU. They found that depression, anxiety, stress, and self-efficacy improved significantly for participants across both the intervention and control groups, with no significant differences in symptom improvement between control and intervention groups [31]. This suggests that symptom improvement for parents was likely attributable to time and distance from the NICU experience, rather than the intervention.

Taken together, research on emotional support interventions for NICU fathers is somewhat mixed, but generally promising. Findings, methodology, and rigor of the research in this area vary, and further research is needed.

#### 3.2.2. Educational Support Interventions

Educational support interventions, in which information was provided to fathers about the NICU stay and how to care for their medically fragile infant, have been effective at minimizing symptoms of distress among NICU parents. As discussed above, in a sample of 34 fathers (compared with 35 father controls) in Taiwan, an intervention providing education related to the NICU stay and the needs of medically fragile infants was found to have a positive impact on fathers [25,26]. These materials were delivered both in writing and via multiple education sessions with a NICU nurse. Fathers who received educational intervention, as compared with controls, had significantly higher perceived nursing support [26] significantly reduced paternal stress at discharge [25,26], significantly higher reports of fathering ability at discharge [25,26], and significantly higher parent-infant attachment one-month post-discharge as compared with controls [25]. Another study, conducted in Iran, explored the use of an educational program providing information on the general condition of their baby, equipment in the NICU, spouse support strategies, and problem solving strategies, there was a significant reduction in paternal stress in the intervention group compared with the control group at two days after admission, five days after admission, and a week after the intervention, as compared with controls [32].

However, other studies have failed to find added value in paternal mental health. An educational intervention known as “Creating Opportunities for Parent Empowerment” provided audio-recorded and written guidance related to parenting and preterm infant development [33]. Fathers who participated in this intervention were more involved in their infants’ care and had more confidence in their parenting role as compared to controls. However, unlike mothers, fathers did not appear to maintain decreased anxiety and depressive symptoms after NICU discharge [33]. Similarly, a study using a video-feedback intervention to support the parent-infant relationship did not have significant effects on parent wellbeing or stress; however, this intervention was found to have a significantly positive impact on father infant bonding [34]. Finally, a study examining the effectiveness of a five-session family-centered educational support program in Iran found no differences in outcomes between fathers in the control and intervention groups [35]. This study found that parental stress decreased in both intervention and control groups around the time of discharge, suggesting that improvements in infant health, which is marked by discharge from the hospital, may have been the main driver of reductions in stress, and education-based intervention was no more effective than standard of care in this context [35].

While some findings on the effectiveness of education-based interventions for NICU fathers are promising, findings are mixed, and more work is needed, particularly to develop interventions that promote lasting improvements in paternal mental health beyond discharge. Additionally, future work should differentiate the educational components that support paternal mental health in some interventions as compared to the interventions that do not seem to improve paternal mental health.

#### 3.2.3. Social Support Interventions

The Chan & Shorey [15] meta-analysis highlighted that parents’ perceptions of social support from friends and family were insignificant across the multiple studies. However, they identified that studies measuring perceived social support did not include intervention components aimed at bolstering social support from family and friends; rather, they simply assessed this outcome without providing targeted intervention. It remains possible that interventions aimed at helping fathers build social support networks during this time of increased stress and logistical demands could be effective in shifting parents’ perceptions of the value of social support [15].

#### 3.2.4. Family-Integrated Care

Family-integrated care is considered the gold standard in the NICU and involves, at a minimum, frequent parental access to the NICU, encouragement of parent participation in infant caregiving, and open communication between parents and the medical team [36]. Researchers in Denmark developed a “Father-Friendly NICU” in an effort to better integrate fathers into babies’ care and to address paternal needs [37,38]. The framework included staff encouragement and accommodations to promote paternal involvement in infant care and medical updates, parent support groups, extended family involvement, and counseling from social workers regarding paternity leave and economic issues. Interestingly, fathers felt less supported by nursing staff and more stressed following the implementation of the “Father-Friendly NICU” intervention as compared with fathers who had infants in the same NICU prior to its implementation [37,38]. The authors hypothesized that these negative impacts were likely the result of increased pressure from staff, when fathers were already struggling to juggle competing demands such as the need to continue providing financially for their family and caretake for older children at home. These results highlight important differences in the fathering role that may limit the generalizability of interventions designed to support mothers in the NICU.

There are important nuances to consider when providing support to fathers, as well as the need to assess individual differences in family structure, socioeconomic demands, and division of labor. Efforts to support NICU fathers should not be solely focused on increasing paternal involvement in the NICU, but should also acknowledge and support fathers in the context of their logistical demands, family structure, cultural background, and individual situations and goals. Researchers and providers must be aware of their own values and biases, ensuring they do not impose personal values or beliefs on the families they aim to support.

#### 3.2.5. Multi-Component Interventions

Some interventions have been designed to include multiple intervention components, typically combining components of education and emotional. One study used a modified version of the Mother-Infant Transaction Program, a program designed to educate and enhance parent-infant attunement, which was adapted to include an initial session for parents to share their feelings of grief related to their infant’s hospitalization [39]. Within this study, father participation in the intervention was limited, with a median participation in six of the 12 intervention sessions. Despite the lower intervention dosage than intended, results showed that paternal stress, as measured by the Parent Stress Index, was significantly lower in the intervention group as compared with the control group [39].

Another study conducted in Iran found that fathers who participated in an intervention that included educational and peer support components reported decreased stress levels as compared with controls [40,41]. Parents were provided with a 60 min educational session in which they were taught about various aspects of the NICU stay, including staff, equipment, environment, and parental participation, among other topics. They also participated in a 2 h peer support group, where they were given the opportunity to share their emotional experiences with other parents. Researchers found that intervention participants had significantly reduced stress levels as measured by the Parental Stressor Scale-NICU scores as compared with controls [40,41]. Of note, while Abdeyazdan et al. [41] reported results in a combined sample of mothers and fathers, limiting the ability to make conclusions about stress reduction based on gender, Shahkolahi et al. [40] found a decrease in stress specific to fathers within the same cohort [40].

A recently published randomized controlled trial evaluating the “Transition to Home” model, designed to optimize transitional care for families with preterm infants, incorporated components of individual support for parental mental health and education to promote competencies related to the child’s development [42]. Researchers found significantly lower state anxiety in fathers before discharge at infant age 35 weeks gestation, as compared with the control group [42]. However, they did not find significant differences between the intervention and control groups with respect to fathers’ mental health over the first six months at home following the NICU stay, as they did for mothers. Given that the transition home is a high-risk period for paternal mental health, it is crucial to find interventions to support fathers during this time [42].

Finally, researchers in Spain found that a five-step, individualized, face-to-face intervention implemented by a psychologist showed significantly lower levels of anxiety symptoms in fathers who received the intervention as compared with controls at day 15 of the NICU admission, and significantly lower levels of depression symptoms at the time of discharge [43]. This promising intervention included providing introductory details to the NICU and the infant’s condition, integration with other parents, guidance in interpreting baby’s signals, psychoeducation about stress, anxiety, and grief, and planning for discharge, amongst other topics and supports. In future work, it will be valuable to assess which components of the combined intervention approaches produce the biggest improvement for paternal mental health.

#### 3.2.6. Summary of Interventions

While initial work is promising, there are major limitations present in the current literature exploring support for NICU fathers. Evidence for the effectiveness of interventions aimed at supporting paternal mental health in the context of NICU is still quite limited, and results are mixed. Interventions developed to target paternal mental health vary widely, and the effective components of studies finding reductions in mental health symptomatology need to be more clearly identified. Further, research must identify interventions that support paternal mental health during the NICU stay, but have lasting impacts throughout the first year postpartum, when NICU fathers are at heightened risk for mental health concerns. Moreover, studies have been conducted across a variety of countries with vastly different cultural values, healthcare systems, and policies, limiting our understanding of the most effective interventions in the United States. It is essential to consider how system structure, policy, multidisciplinary training, and implementation protocols impact the quality of care provided for NICU fathers.

## 4. Challenges and Constraints

### 4.1. Systemic Challenges

Additional aspects that complicate support for NICU fathers are systemic. At the societal level, pressures based on gender norms and expectations produce competing demands for NICU fathers. As highlighted by Risanger et al. [37], fathers are increasingly recognized as important infant caregivers, yet also harbor expectations to be strong, support their partner, care for older children, and provide financially. These responsibilities are not only reinforced socially, but also on a political level, with limited to no universal paternity level policies currently existing in many countries. Research has shown that adequate paternity leave not only impacts paternal mental health [44], but also impacts maternal and family wellbeing [45]. Thus, societal expectations and the current political landscape are major barriers to father and family wellbeing.

There are also hospital-system-based challenges that limit the support and involvement of fathers in the NICU. For example, some hospital have strict visiting policies, including limited visiting hours which may substantially limit working fathers’ abilities to spend time with their infants [3]. Some NICUs only allow one parent in the unit at a time. Often, families opt to have the mother be the primary visitor given biological needs such as breastfeeding. Even when there are efforts to emphasize family-based care in the NICU, the medical record is set up such that the infant is the identified patient. Thus, documentation and billing of individualized psychological support for NICU parents becomes complicated. While some services can be deemed as targeting the child’s wellbeing via bolstering parent–child bonding, when interventions become more individualized and aimed at identified mental health concerns for the parent, it is no longer appropriate to chart in the infant’s records. Not only is there an obvious mismatch between the patient and record, but privacy concerns also arise with documentation of parental mental health in the child’s medical records, as these records will be accessible to the child and the child’s medical providers. Thus, while integrated mental health support in the NICU is ideal, mental health providers typically need to have an affiliation with another department providing interventions for adults in order to provide an adequate level of mental health care to NICU parents. This is particularly challenging in NICUs that are part of a children’s hospital, where adults are not taken as patients. While some mental health providers are associated with obstetrics departments, in which the birthing parent is the identified patient, this still leaves fathers out.

There are rare yet promising policies that are being implemented in some states in the United States. For example, California has created payment structures for dyadic services and family therapy [46]. It allows for reimbursement for screening of parent mental health and services for parent education and maternal mental health. This is a good step in recognizing the importance of caregiver mental health on children and families; however, it still tends to focus on mothers. Moreover, this has only recently been implemented across the state, and organizational structures are still figuring out how to implement the benefit; thus, efforts to evaluate the impact of this policy change are still years away.

An additional consideration in this area is the support of all non-birthing parents, regardless of gender identity. With increased awareness of the spectrum of gender identities in the United States, hospitals have begun to adopt the label “non-birthing parent” to be inclusive of various gender identities and family structures. While the literature lags behind in using this terminology, it is important to recognize that not all non-birthing parents are male or identify as fathers. Moving forward, it is vital that all non-birthing parents, regardless of gender identity, be considered in their need for support.

Future efforts need to be made to address systemic problems, determine sustainable and appropriate funding sources for NICU mental health, and streamline opportunities for mental health care of NICU families, particularly for NICU fathers. The 2023 U.S. Surgeon General’s Advisory [47] on the mental health and wellbeing of parents highlights the increasing recognition of the importance of parental mental health in society, and the stressors identified in this report are particularly intense for NICU parents [48]. It is imperative that fathers are considered in societal efforts to improve family mental health.

### 4.2. Continued Intervention Post-Discharge

Research shows that while mothers’ mental health appears to improve over the course of the NICU stay, fathers’ mental health challenges remain, and the transition home from the NICU may be a time of particular vulnerability for fathers [10,11]. Research suggests that interventions focused on the mother-father-infant triadic relationship, post-discharge, improve long-term parenting stress for fathers, with significant reductions in stress at 18 months postpartum as compared with controls [49]. However, as discussed earlier, the study of the “Transition to Home” model did not find significant improvement in father mental health during the first six months home from the NICU in the intervention group, as compared to the control group, as they did for mothers [42]. Interestingly, the authors note that they may have been limited in their ability to identify effects due to higher dropout rates of fathers. Qualitative work revealed that fathers dropped out of the support program more frequently because they felt less intensely involved and cared for, and they experienced multiple other family burdens that taxed their time [42]. This poses an ongoing challenge for research on fatherhood. Researchers must continue to make an effort to identify interventions not only aimed at supporting fathers during their NICU stay, but also during the transition home.

### 4.3. Training Across NICU Disciplines

To best support fathers, collaboration across NICU disciplines is essential. Gaps in the identification of fathers in need of mental health support remain, and there is a clear need for universal depression screening for not only NICU mothers, but also NICU fathers [11,50]. This requires training for all NICU staff in recognizing the signs of mental health concerns and how these signs may look different for fathers [3]. NICU staff members, particularly nursing staff, are in a unique position to develop strong relationships with parents over the course of the baby’s NICU stay. Given the importance of rapport and the in-depth knowledge nurses have regarding the challenges NICU families face, NICU nurses are well-positioned to provide parents with support [51]. Nurses and other family-facing NICU professionals must be equipped with basic mental health support skills and be offered education on providing trauma-informed care for families. Studies have shown that when properly trained, nurses feel confident in their abilities to implement support for fathers [52]. The recommendation to train and involve nursing staff to provide family support has been echoed by others in the field [50], and a resource with evidence-informed strategies that may be helpful for nurses and other NICU professionals has been created as a guide to support fathers [53].

## 5. Limitations

Because the initial purpose of this literature search was to inform clinical planning, we did not establish a prospective plan for this search and thus did not conduct this as a systematic or scoping review. This is a significant limitation of this project, as there are relevant works that may not have been highlighted in this manuscript. Valuable systematic and scoping reviews published on related topics can be found in other published works, including Hull et al. [3], Chan & Shorey [15], Holm et al. [17], and Ocampo et al. [18]. Additionally, it’s important to note that the groupings of interventions discussed in this manuscript were based roughly on the intervention groupings outlined in Ocampo et al. [18] and were not derived through systematic or statistical analyses within our work.

## 6. Conclusions

It is critical that more work be done to assess and develop interventions to provide psychological support to fathers in the NICU. While some promising interventions for NICU mothers may also be helpful for fathers, it is essential that further interventions be tested directly with fathers, given the emotional, biological, and societal differences fathers face. Moreover, because fathers are at particular risk for mental health concerns post-discharge [11], efforts to continue to support and monitor paternal mental health following infant hospitalization are essential when considering the development of interventions to support NICU fathers. Because traditional healthcare structures support the baby as the identified patient in the NICU, and the mother as the patient in obstetrics and gynecology, additional funding and service streams are needed to engage and support fathers during the perinatal period. Efforts should be made to develop interventions that offer support sensitive to the unique challenges and competing demands of fathers, taking into consideration their cultural background and the impact of system-level policies.

## Data Availability

No new data were created or analyzed in this study.

## Abbreviations

The following abbreviations are used in this manuscript:

NICU	Neonatal Intensive Care Unit
